# CD4^+^ T cell memory is impaired by species-specific cytotoxic differentiation, but not by TCF-1 loss

**DOI:** 10.3389/fimmu.2023.1168125

**Published:** 2023-04-14

**Authors:** Tom Hofland, Luca Danelli, Georgina Cornish, Tiziano Donnarumma, Deborah M. Hunt, Luiz P. S. de Carvalho, George Kassiotis

**Affiliations:** ^1^ Retroviral Immunology Laboratory, The Francis Crick Institute, London, United Kingdom; ^2^ Mycobacterial Metabolism and Antibiotic Research Laboratory, The Francis Crick Institute, London, United Kingdom; ^3^ Department of Infectious Disease, Faculty of Medicine, Imperial College London, London, United Kingdom

**Keywords:** CD4 T cell, granzyme B (GzmB), immunological memory, TCF-1, mouse, human

## Abstract

CD4^+^ T cells are typically considered as ‘helper’ or ‘regulatory’ populations that support and orchestrate the responses of other lymphocytes. However, they can also develop potent granzyme (Gzm)-mediated cytotoxic activity and CD4^+^ cytotoxic T cells (CTLs) have been amply documented both in humans and in mice, particularly in the context of human chronic infection and cancer. Despite the established description of CD4^+^ CTLs, as well as of the critical cytotoxic activity they exert against MHC class II-expressing targets, their developmental and memory maintenance requirements remain elusive. This is at least in part owing to the lack of a murine experimental system where CD4^+^ CTLs are stably induced. Here, we show that viral and bacterial vectors encoding the same epitope induce distinct CD4^+^ CTL responses in challenged mice, all of which are nevertheless transient in nature and lack recall properties. Consistent with prior reports, CD4^+^ CTL differentiation is accompanied by loss of TCF-1 expression, a transcription factor considered essential for memory T cell survival. Using genetic ablation of *Tcf7*, which encodes TCF-1, at the time of CD4^+^ T cell activation, we further show that, contrary to observations in CD8^+^ T cells, continued expression of TCF-1 is not required for CD4^+^ T cell memory survival. Whilst *Tcf7*-deficient CD4^+^ T cells persisted normally following retroviral infection, the CD4^+^ CTL subset still declined, precluding conclusive determination of the requirement for TCF-1 for murine CD4^+^ CTL survival. Using xenotransplantation of human CD4^+^ T cells into murine recipients, we demonstrate that human CD4^+^ CTLs develop and persist in the same experimental conditions where murine CD4^+^ CTLs fail to persist. These observations uncover a species-specific defect in murine CD4^+^ CTL persistence with implications for their use as a model system.

## Introduction

CD4^+^ T cells are classically defined as ‘helper’ populations that can differentiate into several different T helper subsets depending on the immune stimulus and environment (1, 2). Although CD4^+^ T cells were initially thought of as orchestrators of immune responses *via* the production of specific cytokines, more recently a subset of CD4^+^ T cells with cytotoxic potential has been described in both mice and human (3, 4). These CD4^+^ cytotoxic T cells (CTLs) display both lytic granule (via granzyme B (GzmB) and perforin) as well as Fas/FasL mediated cytotoxicity in an MHC class II-dependent manner, and develop especially in the context of prolonged antigen stimulation, such as chronic viral infection and cancer (3–7). Indeed, peptide-specific cytotoxic responses by CD4^+^ CTLs have been described towards multiple viruses and cancers (5, 7–12).

Because CD4^+^ CTLs can provide cytotoxic capacity, additionally to conventional NK cells and CD8^+^ T cells and independently of MHC class I-mediated presentation, there has been a growing interest in including or targeting CD4^+^ CTLs for immunotherapeutic and vaccine strategies. Consequently, since their discovery, there have been several studies that looked into the factors required for CD4^+^ CTL development. It has been shown that loss of the transcription factor ThPOK and upregulation of Runx3 can drive CD4^+^ CTL differentiation (13–15). This transcriptional reprogramming resembles that of CD8^+^ versus CD4^+^ lineage commitment during T cell development and induces expression of more genes classically involved in cytotoxic CD8^+^ T cell differentiation, including CD8α, T-bet, Eomes, and GzmB (13–16). Other factors that have been shown to stimulate CD4^+^ CTL differentiation include signaling *via* 4-1BB, OX40, and IL-2 receptors (17–21). In contrast, the T follicular helper (Tfh) differentiation program antagonizes CD4^+^ CTL differentiation (via upregulation of the transcription factor Bcl6), as does signalling mediated by the inhibitory receptors PD-1 and LAG3 (16).

Despite these studies that looked into the priming of CD4^+^ CTLs, there is less known about the longevity and memory formation of this population. In CD8^+^ T cells, cytotoxic differentiation is antagonistic with longevity and memory formation (22, 23). The transcription factor TCF-1 has been shown to be essential for CD8^+^ T cell memory formation, and the downregulation of TCF-1 in cytotoxic CD8^+^ T cell effector cells reduces their longevity and survival (24). Since CD4^+^ CTLs share some transcriptional programming with CD8^+^ T cells, including loss of TCF-1 expression, it is theoretically possible that induction of a cytotoxic program antagonizes the long-term survival of CD4^+^ CTLs in a similar fashion. A better understanding of the longevity and maintenance of CD4^+^ CTLs is required to determine their immunotherapeutic potential.

Here, we studied the long-term survival of cytotoxic CD4^+^ T cells during chronic infection. We report that GzmB^+^ CD4^+^ T cell populations are not stable in mice, and that cytotoxic differentiation hampers long-term persistence of CD4^+^ T cells. Surprisingly, we found that continued expression of the transcription factor TCF-1 plays no role in either memory formation or the persistence of CD4^+^ T cells. Finally, we found human CD4^+^ CTLs show a higher degree of long-term persistence, indicating a species-specific difference in CD4^+^ CTL survival.

## Materials and methods

### Mice

Inbred C57BL/6J (B6), CD45.1^+^ congenic B6 (B6.SJL-*Ptprca Pep3b*/BoyJ) and *Rag2* ^-/-^ *Il2rg* ^-/-^ *Cd47* ^-/-^ compound deficient mice were originally obtained from The Jackson Laboratory (Bar Harbor, ME, USA). TCRβ-transgenic EF4.1 mice (25), Rag1-deficient (*Rag1* ^-/-^) mice (26), Rag2-deficient (*Rag2* ^-/-^) mice (27), GzmB-tdTomato reporter mice (28), *Tnfrsf4^Cre^* mice (29), *Gt(ROSA)26Sor* ^YFP^ (*R26* ^YFP^) reporter mice (30), and mice with a Cre-conditional *Tcf7* allele (*Tcf7* ^tm1c(EUCOMM)Wtsi^), referred to here as *Tcf7* ^fl/fl^ mice (31), were previously described and were maintained on the B6 genetic background at the Francis Crick Institute’s animal facilities. Mice with activation-induced deletion of *Tcf7* in antigen-specific CD4^+^ T cells were obtained by combining the EF4.1, *Tnfrsf4^Cre^*, *R26* ^YFP^ and *Tcf7* ^fl/fl^ alleles, in a compound mutant strain. All animal experiments were approved by the ethical committee of the Francis Crick Institute, and conducted according to local guidelines and UK Home Office regulations under the Animals Scientific Procedures Act 1986 (ASPA).

### CD4^+^ T cell adoptive transfer

Single-cell suspensions were prepared from the spleens of donor CD45.1^+^ or CD45.2^+^ TCRβ-transgenic EF4.1 mice and CD4^+^ T cells were enriched using an immunomagnetic positive selection kit (StemCell Technologies, Vancouver, Canada), at >90% purity. Donor transgenic CD4^+^ T cells (1×10^6^ per recipient) were injected into recipient mice intravenously.

### Retroviral infection and immunisation

The Friend virus (FV) used in this study was a retroviral complex of a replication-competent B-tropic F-MLV (F-MLV-B) and a replication-defective spleen-focus forming virus (SFFV). Stocks were prepared as previously described (32). Mice were injected intravenously with 0.1 mL PBS containing 1,000 spleen focus-forming units (SFFU) of FV. Ad5.pIX-gp70 stocks were prepared at a titer of 9×10^9^ viral genomes ml^-1^ by infection of 293A cells as previously described (33). Approximately 5×10^8^ Ad5.pIX- gp70 viral genomes per mouse were administered intravenously. Recombinant *Mycobacterium bovis* bacille Calmette-Guérin (BCG) was generated by inserting the sequence encoding the F-MLV gp70 glycoprotein env_122-141_ epitope into the *sodA* gene, using previously described methodology (34). BCG.SOD-env_122-141_ was administered intravenously. Immunization with FBL-3 tumor cells was carried out by intravenous injection of 1.5×10^6^ FBL-3 cells (35). For peptide immunization, mice received an intraperitoneal injection of a total of 12.5 nmol of synthetic env_122-141_ peptide mixed in Sigma Adjuvant System (Sigma-Aldrich, St. Louis, Missouri, USA).

### Plasmids, transfections and retroviral transductions

The pMX plasmid encoding constitutively active STAT5a protein was kindly provided by Ben Seddon. Transfection of the Platinum-E retroviral packaging cell line and retroviral transduction of CD4^+^ T cells were performed as described previously (36).

### Antibodies and flow cytometry

Spleen and lung single-cell suspensions were stained for 20 min at room temperature or at 4^°^C with directly conjugated antibodies to surface markers. For detection of intracellular antigens, subsequent to surface staining, cells were fixed and permeabilized using the Foxp3/Transcription Factor Staining Buffer Set (Thermo Fisher Scientific, Waltham, Massachusetts, USA) according to the manufacturer’s instructions. They were then incubated for 45 min at room temperature with directly conjugated antibodies to intracellular antigens. Zombie UV Fixable Viability Kit (BioLegend, San Diego, California, USA) or Fixable Live/Dead Near IR Dead Cell Stain kit (Invitrogen, Waltham, Massachusetts, USA) was used to label and exclude dead cells from analysis. The following anti-mouse antibodies were used: BV785-, BUV395- or BV711-anti-CD4 (clone GK1.5), PE/Cy7-, PerCP-Cy5.5-, or eFluor450-anti-CD45.1 (clone A20), PE/Cy7-anti-CD279 (PD-1, clone 29F.1A12), V500-anti-CD44 (clone IM7), BV421- or PerCP-Cy5.5-anti-CD162 (PSGL1, clone 2PH1), PE-anti-Bcl6 (clone K112-91), APC-eFluor-780 or BV711-anti-CD45.2 (clone 104), PE-anti-CD223 (LAG3, clone eBioC9B7W), FITC-, PerCP-Cy5.5-, AF700- or APC-anti-TCRβ (clone H57-597) (from Thermo Fisher Scientific, Waltham, Massachusetts, USA); Alexa(R)488- or Alexa(R)647-anti-TCF1 (clone C63D9) (from Cell Signaling Technology, Danvers, Massachusetts, USA), Alexa Fluor 647-anti-ThPOK (clone T43-94, BD Biosciences). For CXCR5 staining, splenocytes were incubated with biotin rat anti-mouse CXCR5 antibody (clone 2G8, BD Biosciences) at 37^°^C for 25 min, followed by incubation with APC- or PE-streptavidin (BioLegend) for 20 min at room temperature. Multi-colour cytometry was performed on LSRFortessa flow cytometers (from BD Biosciences), and analyzed with FlowJo v10.8.1 (Tree Star Inc., Ashland, OR, USA).

### Statistical analyses

Statistical comparisons were made using SigmaPlot 13.0 (Systat Software Inc., Germany) or GraphPad Prism 9 (GraphPad Software, La Jolla, CA 92037 USA). Parametric comparisons of normally distributed values that satisfied the variance criteria were made by unpaired Student’s *t*-tests or One Way Analysis of variance (ANOVA) tests. Data that did not pass the variance test were compared with non-parametric two-tailed Mann-Whitney Rank Sum tests or ANOVA on Ranks tests. P values are indicated by asterisks as follows: * *p*<0.05; ** *p*<0.01; *** *p*<0.001, **** *p*<0.0001. Data were plotted as the mean ± standard error mean.

## Results

### The GzmB^+^ CD4^+^ T cell response does not form memory

**Figure 1:**
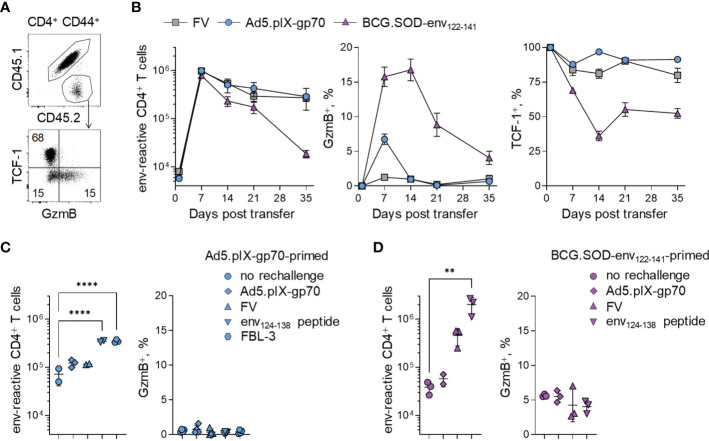
Persistence of env-reactive CD4^+^ CTLs following diverse challenges. **(A)** Flow-cytometric detection of CD44^+^ env-reactive donor CD4^+^ T cells (top) and TCF-1^+^ and GzmB^+^ CD4^+^ T cells (bottom) in spleens of WT recipient mice. **(B)** Absolute numbers (left), GzmB expression (middle) and TCF-1 expression (right) of env-reactive donor CD4^+^ T cells in the spleens of WT recipient mice at indicated time points after FV infection, or immunization with Ad5-pIX-gp70 or BCG.SOD-env_122-141_. **(C)** Absolute numbers (left) and GzmB expression (right) of env-reactive donor CD4^+^ T cells in the spleens of Ad5.pIX-gp70-primed mice at day 7 after re-challenge with indicated stimuli. **(D)** Absolute numbers (left) and GzmB expression (right) of env-reactive donor CD4^+^ T cells in the spleens of BCG.SOD-env_122-141_-primed mice at day 7 after re-challenge with indicated stimuli. **p < 0.01; ****p < 0.0001.

We have previously described an adoptive transfer system that allows the study of CD4^+^ T cell responses to a dominant H2-A^b^-restricted env_122-141_ epitope within the F-MLV gp70 glycoprotein (16, 33, 37). In order to study CD4^+^ CTL differentiation and memory formation, we followed the response of CD4^+^ T cells to FV and various vectors of the env_122-141_ epitope, including Ad5.pIX-gp70 and BCG (which causes persistent infection). CD4^+^ CTLs were identified by staining for GzmB expression and separated from CD4^+^ Tfh counterparts by co-staining for TCF-1 expression (**Figure 1A**). We had previously demonstrated that GzmB^+^ CD4^+^ T cells generated following FV infection or Ad5.pIX-gp70 immunization in this model lose TCF-1 expression and exhibit GzmB-mediated cytotoxic activity against MHC class II-expressing targets, whereas CD4^+^ T cells that retain TCF-1 expression bear all the hallmarks of Tfh cells, including co-expression of CXCR5, BCL6 and PD-1, lack of PSGL1 and SLAM expression, and localization in B cell follicles and germinal centers (16, 38, 39). All immunizations gave rise to a GzmB^+^ CD4^+^ T cell population of varying sizes in the spleens of recipient mice, but in all cases this population was quickly lost in the weeks following the peak of the response (**Figures 1A, B**). BCG induced the biggest GzmB^+^ CD4^+^ T cell population and the most pronounced and persistent loss of TCF-1 in all CD4^+^ T cells, which was evident in ~50% of the cells in the chronic phase of infection (**Figure 1B**). Nevertheless, only 5% of EF4.1 CD4^+^ T cells expressed GzmB in the chronic phase of BCG infection (**Figure 1B**). The loss of GzmB^+^ CD4^+^ T cells from the spleens could not be explained by migration of GzmB^+^ CD4^+^ T cells to effector sites as similar loss of GzmB^+^ CD4^+^ T cells was observed within env-reactive CD4^+^ T cells also in the lung of FV and BCG infected mice (**Figure S1**).

The lack of GzmB in memory CD4^+^ T cell populations could result either from loss of GzmB expression over time within memory CD4^+^ T cells, or the specific loss of GzmB^+^ CD4^+^ T cells themselves. To differentiate between the two possibilities, we rechallenged Ad5-pIX-gp70 or BCG.SOD-env_122-141_ immunized mice using different stimuli. We employed both homologous and heterologous rechallenges, including env_122-138_ peptide-based immunization, as heterologous rechallenges would not be affected by prior CD8^+^ T cell or antibody mediated immunity to the vectors and would recreate the priming environment. Although some stimuli induced bigger CD4^+^ T cell recall expansions, none of the rechallenges gave a GzmB recall response (**Figures 1C, D**, **S2**), indicating that a GzmB^+^ CD4^+^ T cell memory population had not formed following primary immunization.

**Figure 2:**
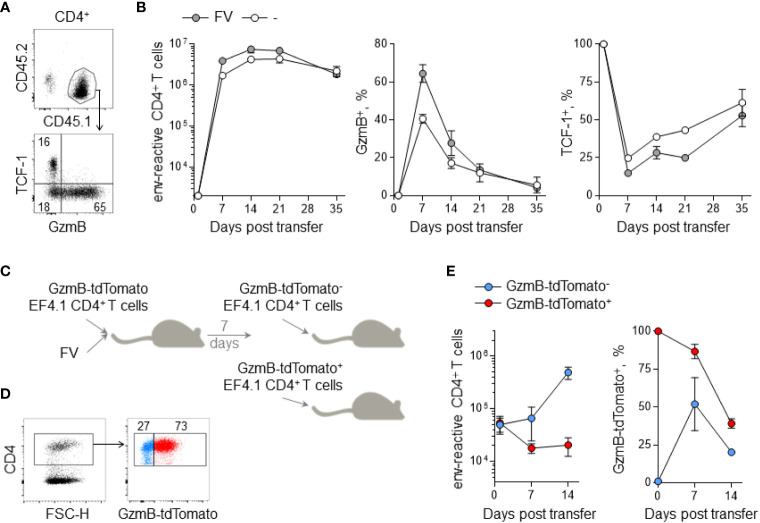
Persistence of env-reactive CD4^+^ CTLs in lymphocyte-deficient hosts. **(A)** Flow-cytometric detection of CD44^+^ env-reactive donor CD4^+^ T cells (top) and TCF-1^+^ and GzmB^+^ CD4^+^ T cells (bottom) in spleens of *Rag1* ^-/-^ recipient mice. **(B)** Absolute numbers (left), GzmB expression (middle) and TCF-1 expression (right) of env-reactive donor CD4^+^ T cells in the spleens of *Rag1* ^-/-^ recipient mice at indicated time points after adoptive transfer with or without FV infection. **(C)** EF4.1 GzmB-tdTomato CD4^+^ T cells were adoptively transferred into FV-infected *Rag1* ^-/-^ recipient mice. After 7 days, donor CD4^+^ T cells were sorted into either GzmB-tdTomato^-^ or GzmB-tdTomato^+^ fractions and injected into new *Rag1* ^-/-^ recipient mice. **(D)** Flow-cytometric detection of GzmB-tdTomato^+^ donor CD4^+^ T cells for cell sorting. **(E)** Absolute numbers (left) and GzmB expression (right) of env-reactive donor CD4^+^ T cells in the spleens of *Rag1* ^-/-^ recipient mice at indicated time points after secondary adoptive transfer.

To test more directly whether GzmB^+^ CD4^+^ T cells can seed a memory population, we transferred EF4.1 T cells into FV infected or uninfected *Rag1* ^-/-^ mice, deficient in T and B cells. *Rag1* ^-/-^ recipient mice that were not deliberately infected with FV carried related infectious murine leukemia viruses (MLVs), arising from recombination of defective endogenous retroviruses (40). These naturally occurring MLVs can also activate the EF4.1 T cells, albeit with lower avidity (41). In this setting, the reactive CD4^+^ T cells generated a very strong CTL response, with ~40% and ~60% of them producing GzmB at the peak of the response in FV infected or uninfected *Rag1* ^-/-^ recipients, respectively (**Figures 2A, B**). This heightened CTL differentiation seen in *Rag1* ^-/-^ recipients was due to the absence of competing CD8^+^ T cells and suppressive Treg cells, as previously described (39, 42). Nevertheless, similarly to those in WT recipients, GzmB^+^ CD4^+^ T cells disappeared over time in *Rag1* ^-/-^ recipients, despite persistent infection and TCF-1 loss (**Figures 2A, B**). To follow the fate of GzmB^+^ effector CD4^+^ T cells, we used a GzmB-tdTomato reporter to mark EF4.1 T cells (16, 28). Following transfer into primary FV-infected *Rag1* ^-/-^ recipients, GzmB-tdTomato^+^ and GzmB-tdTomato^-^ effector CD4^+^ T cells were isolated at the peak of the response and re-transferred into secondary FV-infected *Rag1* ^-/-^ recipients (**Figure 2C**). Transferred GzmB-tdTomato^+^ CD4^+^ T cells showed no secondary expansion in secondary recipients and gradually lost GzmB expression over time. In contrast, GzmB-tdTomato^-^ CD4^+^ T cells expanded considerably after transfer and gave a transient CTL response that was lost again over time, following kinetics similar to the primary response (**Figures 2D, E**). These results suggested that acquisition of the cytotoxic program by CD4^+^ T cells occurs only during the effector response and is incompatible with long-term persistence.

### CD4^+^ CTL differentiation, but not stability is boosted by IL-2 signalling

**Figure 3:**
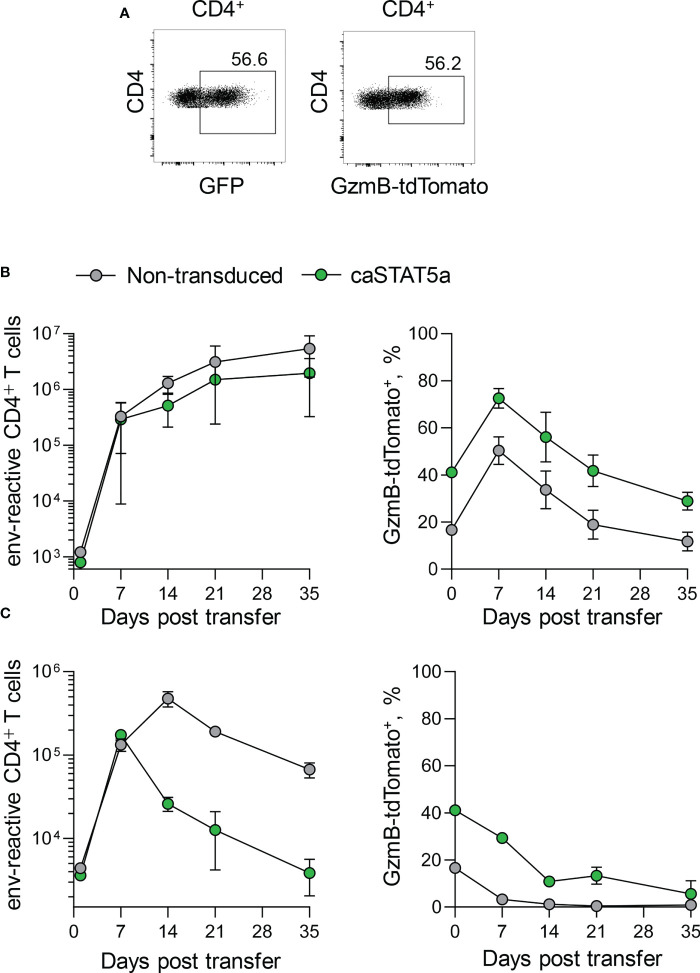
STAT5-dependence of GzmB expression in CD4^+^ T cells. **(A)** Flow-cytometric detection of GFP^+^ caSTAT5a-transduced (top) and GzmB-tdTomato^+^ (bottom) env-reactive donor CD4^+^ T cells in spleens of *Rag1* ^-/-^ recipient mice. **(B)** Absolute numbers (left) and GzmB-tdTomato expression (right) of non-transduced and caSTAT5a-transduced env-reactive donor CD4^+^ T cells in the spleens of *Rag1* ^-/-^ recipient mice at indicated time points after adoptive transfer. **(C)** Absolute numbers (left) and GzmB-tdTomato expression (right) of non-transduced and caSTAT5a-transduced env-reactive donor CD4^+^ T cells in the spleens of WT recipient mice at indicated time points after adoptive transfer.

Expression of the cytotoxic program by CD4^+^ T cells during the effector but not the memory phase of the response to the diverse antigenic stimuli we have used here indicated that this program may rely on factors that were available only during the effector response. One such factor is IL-2, which is produced by effector CD4^+^ T cells. Indeed, IL-2 signaling has been reported to promote CD4^+^ CTL differentiation (20, 21). Furthermore, IL-2 signalling during CD4^+^ T cell priming has been shown to play an important role in memory formation (43, 44). Consistent with published reports, stimulation of GzmB-tdTomato EF4.1 T cells with IL-2 and IL-12 led to substantially increased GzmB expression (**Figure S3A**). It was therefore possible that reduced GzmB production at later time-points during the *in vivo* response or during recall responses was due to low IL-2 availability. To test if IL-2 signaling could increase the lifespan of GzmB^+^ CD4^+^ T cells, we transduced GzmB-tdTomato reporter EF4.1 T cells with a plasmid vector encoding constitutively active STAT5a (caSTAT5a) to mimic continuous IL-2 signaling. In FV infected *Rag1* ^-/-^ recipients, caSTAT5a-expressing CD4^+^ T cells showed similar expansion dynamics but increased GzmB expression compared to non-transduced CD4^+^ T cells at all time-points (**Figures 3A, B**). However, despite consistently inducing higher levels of GzmB expression compared to non-transduced cells, GzmB expression in caSTAT5a-expressing CD4^+^ T cells was lost over time with similar kinetics to non-transduced CD4^+^ T cells (**Figure 3B**). Similar results were obtained in WT recipient mice, although caSTAT5a-expressing CD4^+^ T cells were specifically lost over time compared to non-transduced CD4^+^ T cells (**Figures 3C**, **S3B, E**). These results indicated that IL-2 signalling boosted CD4^+^ CTL differentiation and GzmB expression, but it did not increase the stability or lifespan of GzmB^+^ CD4^+^ T cells.

### CD4^+^ CTL kinetics are independent from CD4^+^ memory differentiation requirements

An alternative explanation for the transient nature of the CD4^+^ CTL response we observed is that this transcriptional program requires loss of transcription factors thought to be required for CD4^+^ memory differentiation, such a ThPOK and TCF-1. Indeed, ThPOK downregulation has been shown to be associated with CD4^+^ CTL differentiation, and ThPOK deficient CD4^+^ T cells express more GzmB (13–15). However, loss of ThPOK is also associated with reduced memory formation in CD4^+^ T cells (45). Therefore, if loss or reduction in ThPOK expression were required for CD4^+^ CTL development, it could also compromise memory formation by GzmB^+^ CD4^+^ T cells in our model. To investigate if reduced ThPOK expression specifically in GzmB^+^ CD4^+^ T cells could underlie their reduced persistence, we measured ThPOK expression in EF4.1 CD4^+^ T cells after adoptive transfer in FV infected *Rag1* ^-/-^ mice by flow cytometry. At the peak of the CD4^+^ T cell response (day 7), we observed no difference in ThPOK expression between TCF-1^+^ and GzmB^+^ CD4^+^ T cells (**Figure S4**). Lack of ThPOK downregulation in this system indicated that reduction in ThPOK expression was neither required for the development of GzmB^+^ CD4^+^ T cells nor could it account for their transient nature.

### TCF-1 expression post activation is dispensable for CD4^+^ T cell memory

GzmB^+^ CD4^+^ T cells are characterized by a number of transcriptional changes (13, 15, 16). These include the acquisition of CD8^+^ T cell markers, and the loss of TCF-1 (encoded by *Tcf7*). Although a role for continuous *Tcf7* expression for T cell memory formation has not been investigated in CD4^+^ T cells at the level of detail that has been for CD8^+^ T cells, it is suggested in at least two experimental settings (46) and maintenance of TCF-1 expression in effector CD4^+^ T cells has been linked with enhance self-renewal capacity (47). It was therefore plausible that, firstly, TCF-1 was necessary for survival of all CD4^+^ memory T cells and, secondly, its loss specifically in GzmB^+^ CD4^+^ T cells was responsible for their reduced persistence into memory.

In order to investigate the role of TCF-1 in GzmB^+^ CD4^+^ T cells, we conditionally deleted *Tcf7* upon CD4^+^ T cell activation using mice with a Cre-conditional *Tcf7* allele (*Tcf7* ^fl/fl^) crossed with mice expressing Cre under the control of the *Tnfrsf4* locus (*Tnfrsf4* ^Cre^), referred to here as *Tcf7* activation-induced deletion (*Tcf7* ^AD^) mice. *Tcf7* ^AD^ mice were additionally crossed with mice with a Cre-conditional YFP reporter allele (*R26* ^YFP^ mice) and with EF4.1 TCRβ transgenic mice, to allow tracking of Cre-mediated recombination and antigen-specific CD4^+^ T cells, respectively.

We first analyzed the degree of Cre-mediated recombination and potential effects on CD4^+^ T cell numbers and composition in naïve *Tcf7* ^AD^ mice, using littermates with one functional *Tcf7* allele (*Tcf7* ^fl/wt^) as controls. As previously described for the activity of the *Tnfrsf4* ^Cre^ driver in this system (37), a majority of memory-phenotype CD44^+^CD4^+^ T cells (60-70%) and of CD25^+^ Treg cells (80-90%) expressed YFP, indicating they had expressed *Tnfrsf4*, whereas only a minority of naïve CD44^-^CD25^-^CD4^+^ T cells (<10%) expressed YFP in control *Tcf7* ^fl/wt^ mice (**Figure S5**). Unexpectedly, these percentages were unaltered by deletion of *Tcf7* in *Tcf7* ^fl/fl^ mice, arguing against an essential role for TCF-1 in maintaining the activated populations (**Figure S5**). Indeed, absolute numbers of naïve, memory-phenotype and Treg cells in the spleen or lymph nodes were indistinguishable between *Tcf7* ^fl/fl^ and control *Tcf7* ^fl/wt^ mice (**Figure S5**).

**Figure 4:**
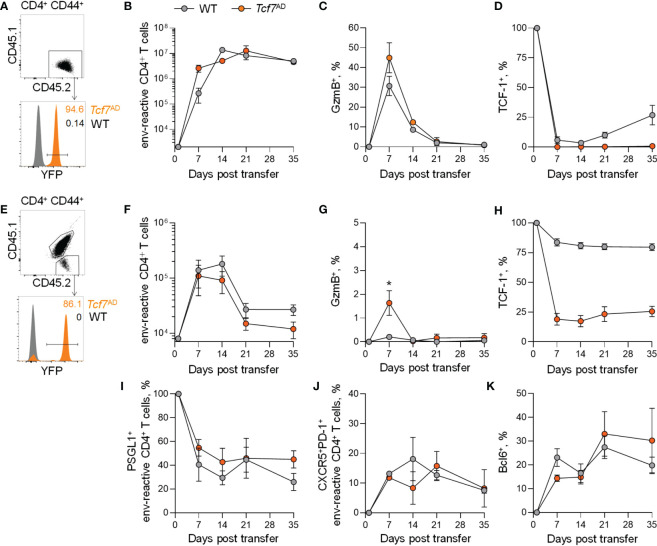
Memory CD4^+^ T cell, but not CTL maintenance independently of TCF-1. **(A)** Flow-cytometric detection of CD44^+^ env-reactive donor CD4^+^ T cells (top) and YFP^+^ CD4^+^ T cells (bottom) in spleens of *Rag1* ^-/-^ recipient mice. **(B)** Absolute numbers of WT EF4.1 and *Tcf7* ^AD^ EF4.1 env-reactive donor CD4^+^ T cells in the spleens of FV-infected *Rag1* ^-/-^ recipient mice at indicated time points after adoptive transfer. **(C)** GzmB expression and **(D)** TCF-1 expression of WT EF4.1 and *Tcf7* ^AD^ EF4.1 env-reactive donor CD4^+^ T cells in the spleens of FV-infected *Rag1* ^-/-^ recipient mice at indicated time points after adoptive transfer. **(E)** Flow-cytometric detection of CD44^+^ env-reactive donor CD4^+^ T cells (top) and YFP^+^ CD4^+^ T cells (bottom) in spleens of WT recipient mice. **(F)** Absolute numbers of WT EF4.1 and *Tcf7* ^AD^ EF4.1 env-reactive donor CD4^+^ T cells in the spleens of FV-infected WT recipient mice at indicated time points after adoptive transfer. **(G)** GzmB expression and **(H)** TCF-1 expression of WT EF4.1 and *Tcf7* ^AD^ EF4.1 env-reactive donor CD4^+^ T cells in the spleens of FV-infected WT recipient mice at indicated time points after adoptive transfer. **(I)** PSGL-1 expression of WT EF4.1 and *Tcf7* ^AD^ EF4.1 env-reactive donor CD4^+^ T cells in the spleens of FV-infected WT recipient mice at indicated time points after adoptive transfer. **(J)** Frequency of CXCR5^+^PD-1^+^ and **(K)** Bcl6^+^ cells within total WT EF4.1 and *Tcf7* ^AD^ EF4.1 env-reactive donor CD4^+^ T cells in the spleens of FV-infected WT recipient mice at indicated time points after adoptive transfer. *p < 0.05.

To examine a possible role for TCF-1 in a well-defined cohort of antigen-specific CD4^+^ T cells, we used *Tcf7* ^AD^ EF4.1 CD4^+^ T cells as donors for adoptive transfer into FV-infected recipients. Following transfer into *Rag1* ^-/-^ recipients, *Tcf7* ^AD^ CD4^+^ T cells showed comparable primary expansion, survival and memory formation when compared with WT EF4.1 CD4^+^ T cells, which were used as controls (**Figures 4A, B**).

At the peak of the response, a moderately higher proportion of *Tcf7* ^AD^ than of WT donor EF4.1 CD4^+^ T cells differentiated into CTLs, as evidence by GzmB expression, but this proportion quickly declined thereafter with similar kinetics in both types for donor CD4^+^ T cell (**Figure 4C**). This unexpected finding indicated that TCF-1 does not play an important role for CD4^+^ T cell survival after T cell activation, which is in sharp contrast to the essential role of TCF-1 for the survival of CD8^+^ T cells. The unaffected survival of *Tcf7* ^AD^ EF4.1 CD4^+^ T cells, compared with WT counterparts, was not due to inefficient *Tcf7* deletion or outgrowth of CD4^+^ T cells that has escaped deletion, as *Tcf7* ^AD^ donor EF4.1 CD4^+^ T cells remained negative for TCF-1 protein expression throughout the observation period, during which TCF-1 was re-expressed in a proportion of WT donor EF4.1 CD4^+^ T cells (**Figure 4D**).

Similar results were obtained when WT recipients were used, where donor EF4.1 CD4^+^ T cells would compete for space with host CD4^+^ T cells (**Figures 4E, H**), except smaller numbers of donor cells were recovered past the peak of the response. Although there was a trend of a smaller expansion of *Tcf7* ^AD^ EF4.1 CD4^+^ T cells in WT hosts, this did not reach statistical significance (**Figure 4F**). Despite smaller proportions of donor EF4.1 CD4^+^ T cells producing GzmB in WT hosts, this production was significantly higher in *Tcf7* ^AD^ than in WT EF4.1 CD4^+^ T cells at the peak and quickly declined thereafter (**Figure 4G**). Loss of TCF-1 was less complete in *Tcf7* ^AD^ donor EF4.1 CD4^+^ T cells transferred into WT than in *Rag1* ^-/-^ recipients, owing to reduced overall activation of donor EF4.1 CD4^+^ T cells in the T cell- and Treg-replete environment of WT recipient, but, importantly, we observed no preferential expansion or survival of TCF-1-expressing cells over time even within the *Tcf7* ^AD^ donor EF4.1 CD4^+^ T cell population (**Figure 4H**). This finding again argues against a requirement for TCF-1 for survival of primed CD4^+^ T cells. In addition to comparable persistence, despite higher peak GzmB production by *Tcf7* ^AD^ than by WT EF4.1 CD4^+^ T cells, loss of TCF-1 post activation did not influence overall differentiation of CD4^+^ T cells and the relative ratio of the mutually exclusive CTLs and Tfh subsets over time. Indeed, *Tcf7* ^AD^ CD4^+^ T cells differentiated into CXCR5^+^PD-1^+^PSGL-1^-^ Tfh cells, and upregulated Bcl6 to a similar extent as WT EF4.1 CD4^+^ T cells (**Figures 4I-K**).

These data indicate that TCF-1 does not play a major role in CD4^+^ T cell differentiation and survival after T cell activation, and a lack of TCF-1 expression therefore cannot explain the loss of GzmB^+^ CD4^+^ T cells in the memory phase.

### Human CD4^+^ CTLs show long term persistence

**Figure 5:**
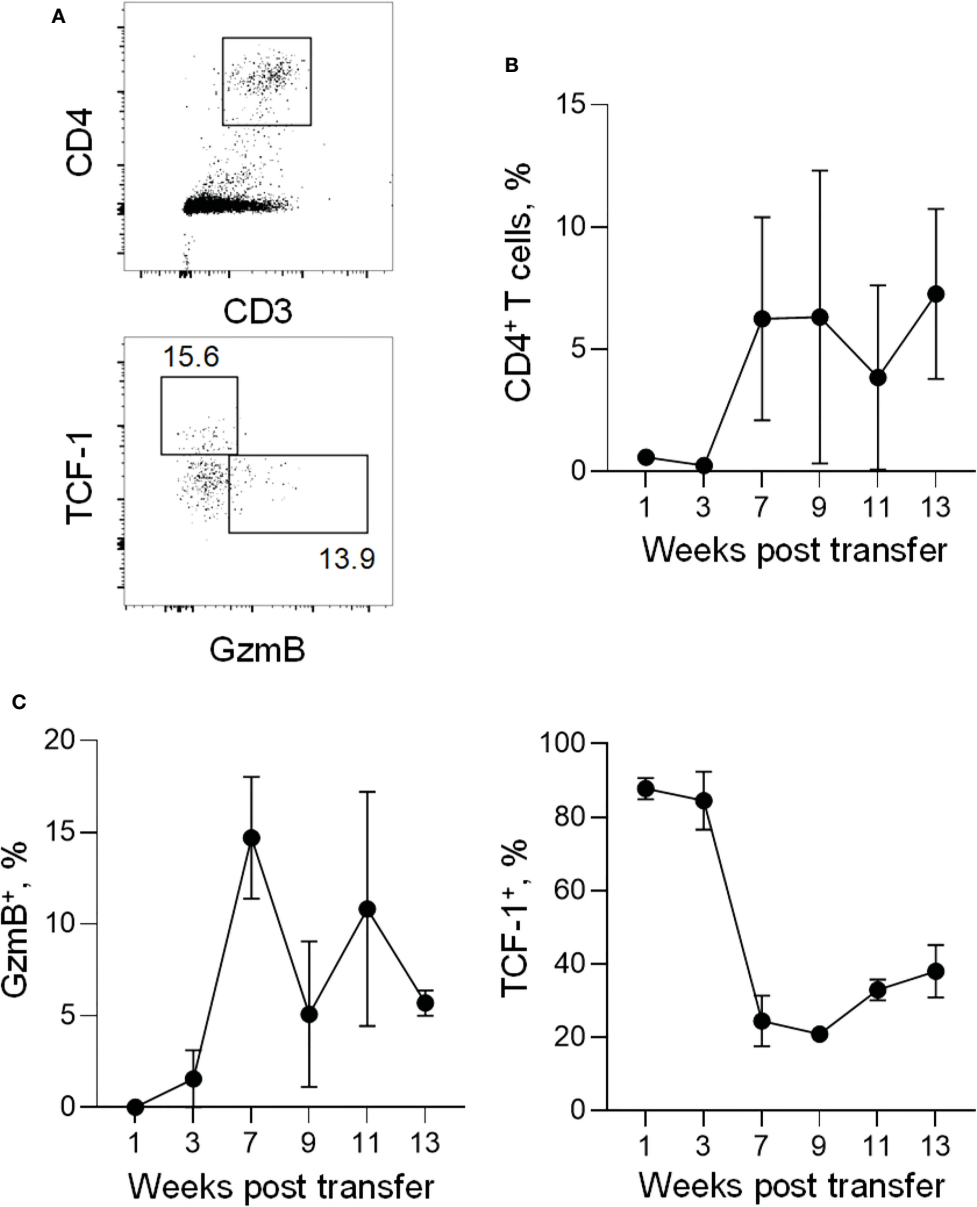
Expansion and persistence of xenografted human CD4^+^ CTLs. **(A)** Flow-cytometric detection of human CD4^+^ T cells (top) and GzmB^+^ and TCF-1^+^ CD4^+^ T cells (bottom) in peripheral blood of recipient triple KO mice injected with human PBMCs. **(B)** Frequency of human CD4^+^ T cells within single cells in peripheral blood (for weeks 1-11) or within splenocytes (week 13) of recipient triple KO mice at indicated time points after adoptive transfer. **(C)** Frequency of GzmB^+^ cells (left) or TCF-1^+^ cells (right) within total human CD4^+^ T cells in peripheral blood (weeks 1-11) or splenocytes (week 13) of recipient triple KO mice at indicated time points after adoptive transfer.

The lack of persistence of murine GzmB^+^ CD4^+^ T cells in our model is in stark contrast to what has been reported for human CD4^+^ CTLs, which have been shown to expand and persist, especially in the context of chronic viral infection (48, 49). This raised the possibility of a species difference in the ability of CD4^+^ CTLs to persist post activation or in settings of chronic antigenic stimulation. To test for a potential difference, we transferred human peripheral blood mononuclear cells (PBMCs) into *Rag2* ^-/-^ *Il2rg* ^-/-^ *Cd47* ^-/-^ compound mutant mice and followed human donor CD4^+^ T cells over time to analyse the persistence of human CD4^+^ CTLs, in the same settings where murine CD4^+^ CTLs failed to persist. Seven weeks after transfer, human CD4^+^ T cells established a stably engrafted population in recipient mice (**Figures 5A, B**). Similar to peak effector murine CD4^+^ T cells, human CD4^+^ T cells developed a population of GzmB^+^ CD4^+^ CTLs that lost expression of TCF-1 (**Figure 5C**). GzmB^+^ CD4^+^ T cells comprised between 5% and 15% of the total human donor CD4^+^ T cells, in line with estimates of the proportion of human CD4^+^ T cells xenoreactive with murine MHC-II molecules (50). These human CD4^+^ CTLs formed a stable population over a period of at least 6 weeks after initial differentiation (**Figure 5C**). This was in contrast to murine GzmB^+^ CD4^+^ T cells, which were lost almost completely after 3-5 weeks of adoptive transfer. The stability of human GzmB^+^ CD4^+^ T cells was intrinsically programmed, as similar results were obtained by adoptive transfer of isolated human CD4^+^ T cells (**Figure S6**). These data indicate there is a species-specific difference in the cytotoxic programming of human and murine CD4^+^ T cells, which prevents long-term persistence specifically in murine CD4^+^ CTLs.

## Discussion

Cytotoxic CD4^+^ T cells have gained considerable interest in the last years as a new immune cell modality that could possibly be targeted for immunotherapeutic strategies. Here, we describe that the cytotoxic differentiation of CD4^+^ T cells antagonizes their longevity and memory formation in the context of chronic infection in mice.

The lack of memory formation by CD4^+^ CTLs could perhaps be a biological mechanism to protect the host from extensive immunopathology. CD4^+^ CTLs have been shown to exacerbate or be involved in multiple autoimmune diseases, including multiple sclerosis (MS) and rheumatoid arthritis (RA) (3, 51). Therefore, reducing the survival of CD4^+^ CTLs could be a strategy to limit the potential damaging effect of this population for the host. Interestingly, long-term effects of CD4^+^ CTL responses are not only limited by reduced survival of these cells, but also by a lack of cytotoxic differentiation of CD4^+^ T cells during recall responses. Although we did not investigate what limits cytotoxic differentiation during secondary stimulation, it could be explained by signalling *via* inhibitory receptors PD-1 and LAG-3, which we have shown are able to inhibit CD4^+^ CTL differentiation (16). Indeed, both PD-1 and LAG-3 are expressed by env-specific CD4^+^ T cells in the memory phase. Furthermore, transcriptional and epigenetic programming of memory CD4^+^ T cells could block the induction of cytotoxic differentiation in secondary immune responses. Similar mechanisms have been described in CD8^+^ T cells, where memory and cytotoxic effectors are characterised by divergent transcriptional and epigenetic states that determine which subset CD8^+^ T cell differentiate into and which prevent the acquisition of cytotoxic potential in memory CD8^+^ T cells (22, 52). Epigenetic programming has already been shown to regulate recall responses of memory CD4^+^ T cells, where memory cells formed by Tfh or Th1 cells induced a recall response of the same initial T cell subset (53). Therefore, lack of CD4^+^ CTL differentiation during recall responses could be explained by CD4^+^ CTL cells not seeding a memory population, which leads to a loss of this subset during a second stimulus. Interestingly, the mechanisms that prevent cytotoxic differentiation of CD4^+^ T cells in the memory phase seem to be very potent, since we show that they cannot be overcome by deliberate chronic activation of the cytotoxic differentiation pathway mediated by constitutive IL-2 signalling, and loss of CD4^+^ CTLs over time is not prevented.

CD4^+^ CTLs are characterized by loss of TCF-1, which is essential for CD8^+^ T cell memory survival, and is also expressed by memory CD4^+^ T cells. We therefore hypothesized that loss of TCF-1 in CD4^+^ CTLs could be responsible for their limited survival. However, here we show that following CD4^+^ T cell activation, loss of TCF-1 does not affect memory CD4^+^ T cell survival. In fact, we show that loss of TCF-1 has no effect at all on CD4^+^ T cells after T cell activation, since we find equal expansion and survival of CD4^+^ T cells, but also similar differentiation into CD4^+^ CTLs and Tfh cells in *Tcf7* ^AD^ mice. Previous studies on the role of TCF-1 in CD4^+^ T cells have shown that TCF-1 is essential for T cell development, Tfh differentiation and response to allogeneic MHC-II (24, 31, 54–56). However, most of these results were obtained using mouse models where TCF-1 was deleted before T cell stimulation. Our results now indicate TCF-1 only plays a role early after CD4^+^ T cell activation and quickly becomes redundant afterwards in the memory phase. This is in sharp contrast with CD8^+^ T cells, and clearly shows that the role of TCF-1 is different in CD4^+^ T cells. Although it is not possible from our experiments to rule out an effect of TCF-1 on the persistence of CD4^+^ CTLs, our observations that TCF-1 is redundant for survival of conventional murine memory CD4^+^ T cells and human CD4^+^ CTLs (which also lack TCF-1) may indicate that the lack of survival of murine CD4^+^ CTLs is unrelated to TCF-1 loss.

The lack of murine CD4^+^ CTL survival is in contrast to the enormous expansions of CD4^+^ CTLs in humans, where these cells persist over long periods of time (3–7, 48, 49). We show here that human CD4^+^ CTLs are able to survive much longer in the same environment as murine CD4^+^ CTLs. Although we did not investigate what causes this difference from a molecular standpoint, immunological differences between mice and men are well documented (including in CD4^+^ T cell responses) (57–59). It is interesting to ponder if survival of CD4^+^ CTLs has evolved in humans due to the beneficial properties of this immune cell population, or whether this quality has been lost in mice due to the possible detrimental effects to the host described above. Nevertheless, if CD4^+^ CTLs are to be used for immunotherapeutic strategies, the ability to survive for extended periods could be important. Indeed, in a recent study in leukemia patients that developed long time tumour remission after CAR therapy, the responsible CAR T cell population was formed almost completely of persisting cytotoxic CD4^+^ T cells (60). These cells showed similarities to the tumour-specific CD4^+^ CTLs that were found in bladder cancer patients (8). Although the anti-tumour response of CAR T cells is not within the natural MHC-II context of CD4^+^ T cells, it does clearly demonstrate the therapeutic potential of CD4^+^ CTLs as effector cells for anti-tumour immunotherapy. Future studies will need to show whether similar anti-tumour responses can be created using CD4^+^ CTLs within their natural MHC-II context, as seems to be the case for bladder cancer.

In conclusion, we show here that murine CD4^+^ CTLs are not able to form a memory population in the context of chronic viral infection. The lack of CD4^+^ CTL memory was not related to the loss ThPOK or TCF-1, and could not be prevented by chronic IL-2 signalling. Human CD4^+^ CTLs showed improved survival compared to their murine counterparts, which suggests that the mouse is not the ideal model to study the biology or the therapeutic potential of CD4^+^ CTLs. The improved survival of human CD4^+^ CTLs and the potential anti-tumor effects of this population indicates CD4^+^ CTLs could be promising targets for immunotherapeutic strategies.

## Data availability statement

The original contributions presented in the study are included in the article/**Supplementary Materials**, further inquiries can be directed to the corresponding author/s.

## Ethics statement

All animal experiments were approved by the ethical committee of the Francis Crick Institute, and conducted according to local guidelines and UK Home Office regulations under the Animals Scientific Procedures Act 1986 (ASPA).

## Author contributions

TH, LD, GC, TD and DH performed the experiments and analyzed the data. LD, and GK supervised the study. TH and GK wrote the manuscript. All authors contributed to the article and approved the submitted version.
