# Supplementary material for: CD4+ T cell memory is impaired by species-specific cytotoxic differentiation, but not by TCF-1 loss
Source: Front Immunol. 2023 Apr 14;14:1168125. doi: 10.3389/fimmu.2023.1168125 (PMC10140371; doi:10.3389/fimmu.2023.1168125)
Supplement: Supplementary file 1 [file DataSheet_1.pdf]

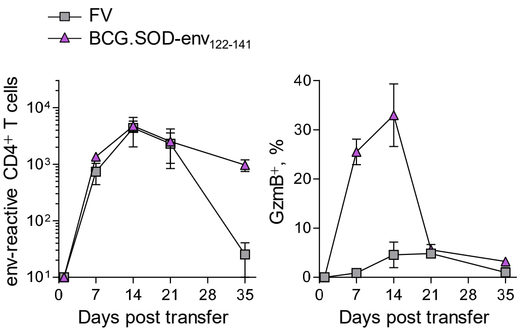

Figure S1. Env-reactive donor CD4$^+$ T cells in the lungs of recipient mice. Absolute numbers (left) and GzmB expression (right) of env-reactive donor CD4$^+$ T cells in the lungs of recipient mice at indicated time points after FV infection or BCG.SOD-env$_{122\text{-}141}$ immunization.

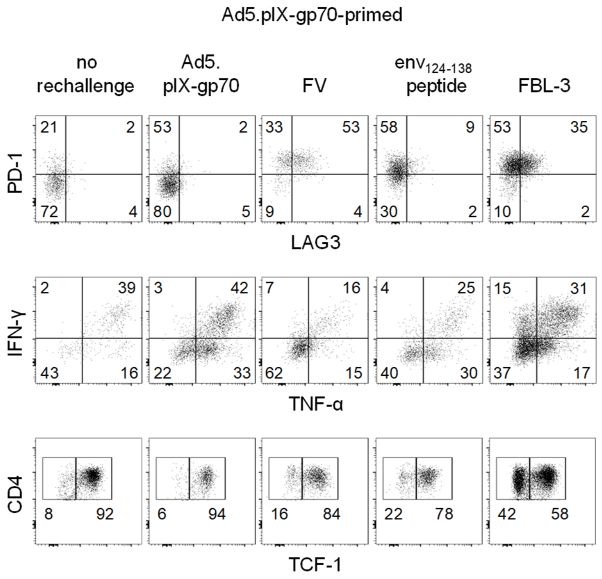

Figure S2. Recall responses of memory env-reactive donor CD4⁺ T cells. Expression of PD-1 and LAG-3 (top row), IFN-γ and TNF-α (middle row) and TCF-1 (bottom row) by env-reactive donor $CD4^+$ T cells in Ad5-pIX-gp70-primed mice 7 days after re-challenge with indicated stimuli.

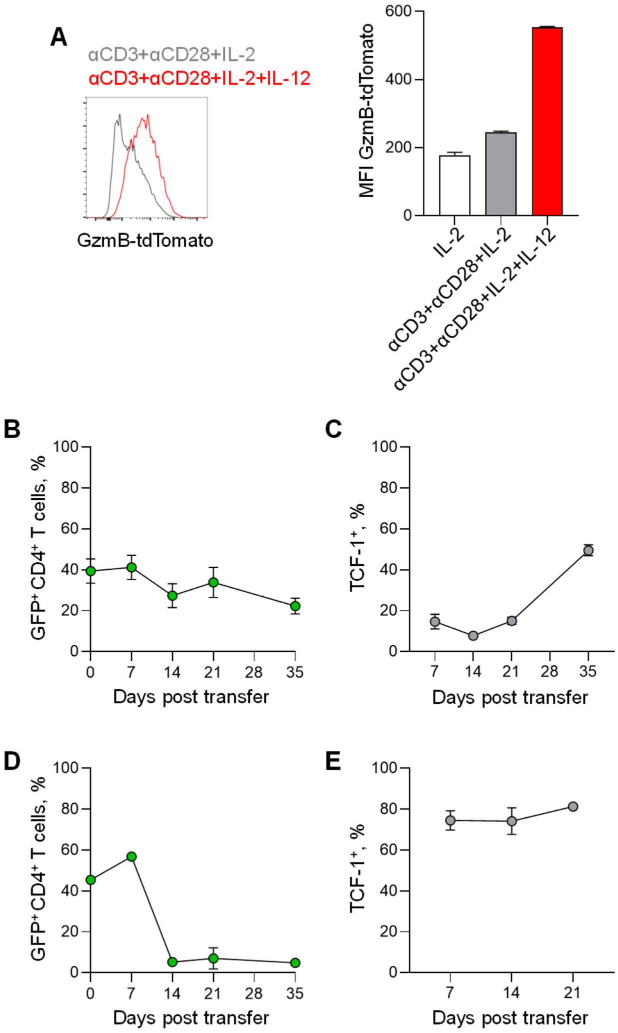

Figure S3. STAT5-dependence of GzmB expression in CD4⁺ T cells. (**A**) Flow-cytometric detection (left) and mean fluorescence intensity (right) of GzmB-tdTomato in env-reactive CD4⁺ T cells stimulated in vitro with anti-CD3, anti-CD28, IL-2 and IL-12. (**B**) Frequency of GFP⁺ caSTAT5-transduced cells within total env-reactive donor CD4⁺ T cells in spleens of FV-infected *Rag1*$^{-/-}$ recipient mice at indicated time points after adoptive transfer. (**C**) Frequency of TCF-1⁺ cells within total env-reactive donor CD4⁺ T cells in spleens of FV-infected *Rag1*$^{-/-}$ recipient mice at indicated time points after adoptive transfer. (**D**) Frequency of GFP⁺ caSTAT5-transduced cells within total env-reactive donor CD4⁺ T cells in spleens of FV-infected WT recipient mice at indicated time points after adoptive transfer. (**E**) Frequency of TCF-1⁺ cells within total env-reactive donor CD4⁺ T cells in spleens of FV-infected WT recipient mice at indicated time points after adoptive transfer.

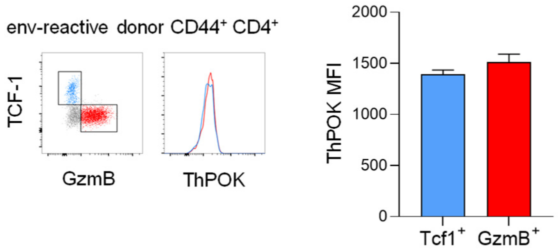

Figure S4. ThPOK expression in env-reactive donor CD4⁺ T cells. Flow-cytometric detection of ThPOK within TCF-1⁺ and GzmB⁺ cells within env-reactive donor CD44⁺ CD4⁺ T cells at day 7 after adoptive transfer into FV-infected *Rag1*$^{-/-}$ recipients (left), and the mean fluorescence intensity of ThPOK in these two populations (right).

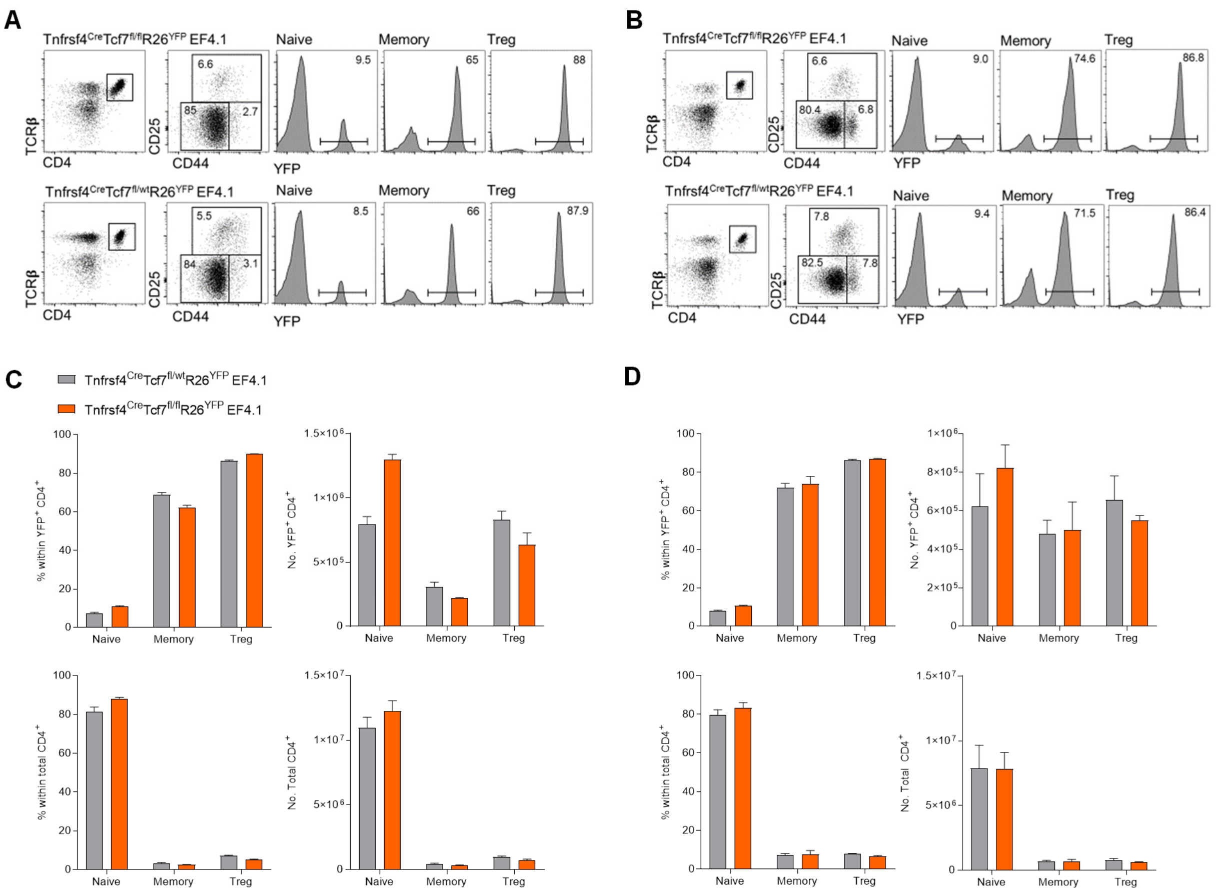

Figure S5. Effect of Tcf7 deletion in naïve and memory CD4$^+$ T cell homeostasis. (**A**) Flow-cytometric detection of CD44$^-$CD25$^-$ naïve, CD44$^+$CD25$^-$ memory and CD44$^+$CD25$^+$ Treg CD4$^+$ T cells, and YFP expression within these subsets in lymph nodes of *Tnfrsf4*^{Cre}*Tcf7*$^{fl/fl}$*R26*^{YFP} EF4.1 and *Tnfrsf4*^{Cre}*Tcf7*$^{fl/wt}$*R26*^{YFP} EF4.1 mice. (**B**) Flow-cytometric detection of CD44$^-$CD25$^-$ naïve, CD44$^+$CD25$^-$ memory and CD44$^+$CD25$^+$ Treg CD4$^+$ T cells, and YFP expression within these subsets in spleens of *Tnfrsf4*^{Cre}*Tcf7*$^{fl/fl}$*R26*^{YFP} EF4.1 and *Tnfrsf4*^{Cre}*Tcf7*$^{fl/wt}$*R26*^{YFP} EF4.1 mice. (**C**) Frequency and absolute number of indicated CD4$^+$ T cell subsets within YFP$^+$ CD4$^+$ T cells (top row) or total CD4$^+$ T cells (bottom row) in lymph nodes of *Tnfrsf4*^{Cre}*Tcf7*$^{fl/fl}$*R26*^{YFP} EF4.1 and *Tnfrsf4*^{Cre}*Tcf7*$^{fl/wt}$*R26*^{YFP} EF4.1 mice. (**D**) Frequency and absolute number of indicated CD4$^+$ T cell subsets within YFP$^+$ CD4$^+$ T cells (top row) or total CD4$^+$ T cells (bottom row) in spleens of *Tnfrsf4*^{Cre}*Tcf7*$^{fl/fl}$*R26*^{YFP} EF4.1 and *Tnfrsf4*^{Cre}*Tcf7*$^{fl/wt}$*R26*^{YFP} EF4.1 mice.

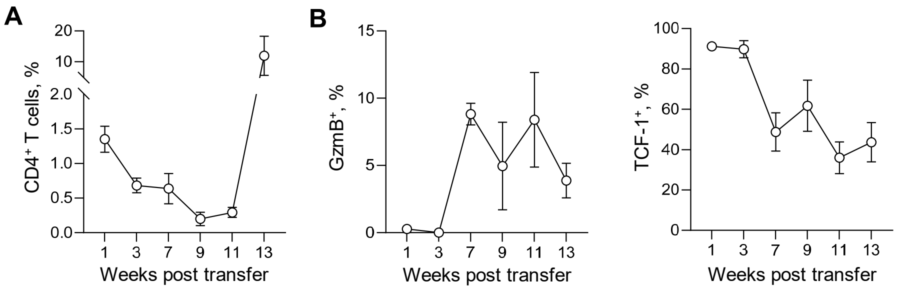

Figure S6. Expansion and Gzmb production by xenografted human CD4$^+$ T cells. (**A**) Frequency of human CD4$^+$ T cells within single cells in peripheral blood (for weeks 1-11) or within splenocytes (week 13) of recipient triple KO mice injected with purified human CD4$^+$ T cells at indicated time points after adoptive transfer. (**B**) Frequency of GzmB$^+$ cells (left) or TCF-1$^+$ cells (right) within total human CD4$^+$ T cells in peripheral blood (weeks 1-11) or splenocytes (week 13) of recipient triple KO mice at indicated time points after adoptive transfer.
